# Simultaneous Analysis of Bacterial and Fungal Communities in Oral Samples from Intubated Patients in Intensive Care Unit

**DOI:** 10.3390/diagnostics13101784

**Published:** 2023-05-18

**Authors:** Yuri Song, Myoung Soo Kim, Jin Chung, Hee Sam Na

**Affiliations:** 1 Department of Oral Microbiology, School of Dentistry, Pusan National University, Yangsan 50612, Republic of Korea; 2 Oral Genomics Research Center, Pusan National University, Yangsan 50612, Republic of Korea; 3 Department of Nursing, College of Natural Science, Pukyong National University, Busan 48513, Republic of Korea; 4 Dental Research Institute, BK21 PLUS Project, School of Dentistry, Pusan National University, Yangsan 50612, Republic of Korea

**Keywords:** microbiota, intubation, mechanical ventilator, *Candida albicans*, NGS, pneumonia

## Abstract

Intubated patients in intensive care units (ICUs) too frequently contract ventilator-associated pneumonia or *Candida* infections. Oropharyngeal microbes are believed to play an important etiologic role. This study was undertaken to determine whether next-generation sequencing (NGS) can be used to simultaneously analyze bacterial and fungal communities. Buccal samples were collected from intubated ICU patients. Primers targeting the V1-V2 region of bacterial 16S rRNA and the internal transcribed spacer 2 (ITS2) region of fungal 18S rRNA were used. V1-V2, ITS2, or mixed V1-V2/ITS2 primers were used to prepare an NGS library. Bacterial and fungal relative abundances were comparable for V1-V2, ITS2, or mixed V1-V2/ITS2 primers, respectively. A standard microbial community was used to adjust the relative abundances to theoretical abundance, and NGS and RT-PCR-adjusted relative abundances showed a high correlation. Using mixed V1-V2/ITS2 primers, bacterial and fungal abundances were simultaneously determined. The constructed microbiome network revealed novel interkingdom and intrakingdom interactions, and the simultaneous detection of bacterial and fungal communities using mixed V1-V2/ITS2 primers enabled analysis across two kingdoms. This study provides a novel approach to simultaneously determining bacterial and fungal communities using mixed V1-V2/ITS2 primers.

## 1. Introduction

Oral bacterial and fungal communities play important roles in various nosocomial infections such as ventilator-associated pneumonia (VAP) [1], hospital-acquired pneumonia (HAP) [2], oral mucositis [3], and systemic infections [4]. In the case of VAP, the microaspiration of oropharyngeal microbes is believed to play a role in the etiology of ventilator-associated pneumonia (VAP) [2], and the prevalence of oral colonization by VAP-associated pathogens such as *Pseudomonas aeruginosa*, *Enterobacteriacea*, *Acinetobacter*, *S. aureus*, and *Streptococcus* spp. are markedly elevated in VAP patients [1]. Moreover, fungal load increases rapidly in intubated patients after admission to intensive care units (ICUs) [5] and can account for a substantial proportion of healthcare-associated infections in intubated patients [6,7]. Proper oral hygiene practices are recommended to reduce the risk of HAIs and other hospital-related conditions caused by oral bacteria and fungus [8].

The monitoring of oral bacterial and fungus abundance changes has several merits. First, the data obtained can be used to predict and diagnose infections [9]. For example, changes in the oral microbiome composition can indicate the presence of infections, such as VAP or bloodstream infections [10]. Second, it provides an understanding of the impact of interventions as a comparison of the oral microbiome before and after interventions, such as antibiotics or oral practice, allows clinicians to understand how these interventions impact the microbiome and potentially enable treatment plans to be adjusted accordingly [11,12]. Thus, the efficient monitoring of oral bacterial and fungal changes in ICU patients and other hospital conditions is important.

Next-generation sequencing (NGS) produces large numbers of sequences at unprecedented speeds. Amplicon analysis is commonly used for microbiome studies and has been used in large projects, including the Human Microbiome Project [13]. The 16S rRNA gene is highly conserved among bacteria and is commonly used to differentiate bacteria at taxonomic levels [14], whereas the internal transcribed spacer (ITS) of the nuclear ribosomal DNA is commonly used in fungal studies. The ITS region is located between the 18S and 28S rRNA genes and comprised of the ITS1 and ITS2 regions, the latter of which is located between the 5.8S and 28S rRNA genes and has been suggested to be useful for clinical studies [15].

In this study, we sought to determine whether bacterial and fungal communities can be simultaneously determined using primers targeting the V1-V2 region for bacteria and the ITS2 region for fungi. Libraries were prepared using individual primers (V1-V2 or ITS2) and combined primers (V1-V2/ITS2 mix) for NGS. The results obtained were compared for alpha diversity and relative abundance at the species level. Furthermore, NGS results were compared with real-time PCR results to confirm the relative abundance measured by NGS.

## 2. Materials and Methods

### 2.1. Patients

The study inclusion criteria applied were: (1) an ICU patient, (2) >18 years of age, (3) endotracheal tube use with a holding method, and (4) consent for participation from the patient’s family. The intubated patients enrolled had a heterogeneous set of underlying diseases (Table S1). Oral samples were collected at multiple time points to monitor microbiome and mycobiome shifts. Broad-spectrum antibiotics were administered to treat underlying diseases. This study was approved by Institutional Review Board (DAUHIRB-19-072). The ICU patients involved were mostly sedated or had poor levels of consciousness, and thus, family members were considered legal representatives with power of attorney as stipulated by the International Council for Harmonization [16]. During the recruitment period, the study purpose, voluntary nature of participation, confidentiality of information, and procedures used in the study were explained to family members, and each family provided informed consent.

### 2.2. Oral Swab Sample Preparation

Buccal samples were collected from 4 patients during ICU treatment (day 0–day 12) using the Levine technique. A sterile cotton swab was rotated over an area of approximately 1 cm^2^ with sufficient pressure [17] and then placed in a conical 15 mL ID-labeled tube and stored at −80 °C.

### 2.3. Extraction of Genomic DNA and Next-Generation Sequencing

Before analysis, samples were suspended in phosphate-buffered saline (PBS), vortexed, and centrifuged. DNA was extracted using a Gram-positive DNA purification kit (Lucigen, Novato, CA, USA) according to the manufacturer’s instructions. Final DNA concentrations were measured using a NanoDrop ND-1000 spectrophotometer (Thermo Fisher Scientific Inc., Waltham, MA, USA), and samples were stored at −80 °C until required. PCR amplification was performed using V1-V2 and ITS2 primer. The primer sequences used for amplifications were as follows: V1-V2-F: 5′-AGA GTT TGA TYM TGG CTC AG-3′, V1-V2-R: 5′-TGC TGC CTC CCG TAG RAG T-3′, ITS2-F: 5′- GCA TCG ATG AAG AAC GCA GC-3′, ITS2-R: TCC TCC GCT TAT TGA TAT GC-3′ [18,19,20]. While 16S rRNA is highly conserved within bacteria, fungal ITS sequences from different species can differ widely in size and sequence content [21]. The theoretical target sizes for the V1-V2 and ITS2 primer pairs were 310 bps and 300–400 bps, respectively. PCR mixes (20 μL) contained 20 ng of the DNA template, primers (20 pmol of each), 200 μM of dNTPs, and 1 unit of Taq DNA polymerase (Zymo, Irvine, CA, USA). Amplification was performed using 24 cycles of 30 s at 94 °C, 30 s at 55 °C, and 30 s at 72 °C, followed by a final 10 min extension at 72 °C. For V1-V2/ITS2 mix PCR, equal concentrations (10 pmol) of V1-V2 primers and ITS2 primers were used. Library was prepared using Herculase II Fusion DNA Polymerase Nextera XT Index Kit V2 (Illumina, San Diago, CA, USA). A ZymoBIOMICS microbial community standard (Zymo, Irvine, CA, USA) was used as the positive control. Purified amplicons were pooled at equimolar concentrations and paired-end sequenced with HiSeq (Illumina, San Diego, CA, USA).

### 2.4. Real-Time PCR

To quantify bacterial and fungal abundance, real-time PCR was applied using species-specific primers for VAP-associated pathogens, including *Acinetobacter baumannii, Pseudomonas aeruginosa, S. aureus, S. epidermidis,* and *C. albicans* (Table S2) [3]. The reaction conditions used for DNA amplification were 95 °C for 10 min followed by 40 cycles of 95 °C for 30 s and 60 °C for 30 s. The analyses were conducted using an RT-PCR system (Thermo Fisher Scientific, Waltham, MA, USA). Total bacteria abundances were determined using universal 16S rRNA, and total fungal abundances using 18S rRNA. The relative abundances of targets were calculated using the ΔΔCt method using 16S rRNA for normalization [3].

### 2.5. Bioinformatic Analysis, Statistical Analysis, and Visualization

Basic microbiome analysis was performed using QIIME2 (version 2020.6) [22] and its associated plugins. DADA2 (Divisive Amplicon Denoising Algorithm 2) was applied to denoise, join the paired reads, and remove the chimera. Choa1 indices, Faith’s phylogenetic diversity (PD) indices, and Shannon’s indices were used to measure alpha diversities. The Mann–Whitney U test was used to determine the significance of alpha diversities. To assign taxonomy to unique representative sequences, we used pretrained Naive Bayes classifier with the eHOMD [23] and UNITE [21] databases. To visualize the internal interactions and further measurement of the microbial community, Sparse Correlations for Compositional data (SparCC) [24] were used to calculate the correlation coefficient with corresponding *p*-value between each two species. The network was then visualized by Cytoscape [25], with the nodes denoting the species and connections representing the existence of correlation.

### 2.6. Data Availability

The raw sequencing data were deposited at NCBI GenBank under BioProject ID PRJNA773666 (BioSample SAMN22503998–SAMN22504007).

## 3. Results

### 3.1. Sequence Preprocessing

A total of 1,323,155 reads (mean ± SD: 65,299 ± 61,068), 981,886 (49,053 ± 40,833), and 2,782,753 (127,081 ± 58,264) were collected using V1-V2 primers, ITS2 primers, and mixed V1-V2/ITS2 primers, respectively. Total read counts obtained using mixed V1-V2/ITS2 primers were significantly higher than those obtained using V1-V2 and ITS2 primers. DADA2 was applied and final nonchimeric read counts were 47,743 ± 43,204, 43,640 ± 33,073, and 98,815 ± 45,122 for V1-V2, ITS2, and mixed V1-V2/ITS2 primers, respectively. Input to nonchimeric read count percentages were 90.25 ± 3.69, 73.80 ± 4.27, and 77.76 ± 4.26 for V1-V2, ITS2, and V1-V2/ITS2 primers, respectively, and minimum nonchimeric read counts were 8696, 4848, and 31,587, respectively (Table 1).

### 3.2. Taxonomy Comparison

**Figure 1:**
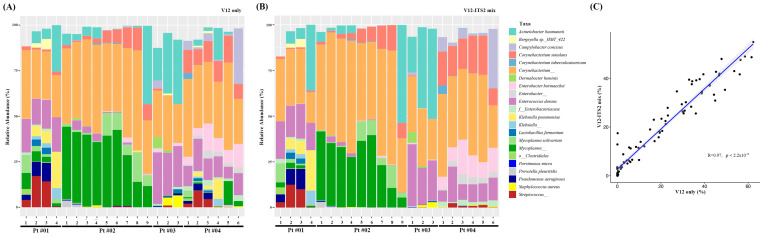
Relative abundances of bacterial species depending on primers: (**A**) V1-V2, (**B**) V1-V2/ITS2 primers, and (**C**) the correlation between abundances determined using V1-V2 and V1-V2/ITS2 primers.

After preprocessing, relative bacterial community abundances obtained using V1-V2 and mixed V1-V2/ITS2 primers were compared. To facilitate the direct comparisons, taxa assigned to the bacteria or fungi kingdom in V1-V2/ITS2 mix data were selected, and relative abundances were calculated for the bacteria and fungi, respectively. Thus, the relative abundance of bacteria from V1-V2/ITS2 was compared with V1-V2 data, and fungal abundance from V1-V2/ITS2 was compared with ITS2 data. When relative abundances were plotted for individual samples depending on the bacterial or fungal kingdom, most taxa showed similar abundances (Figure 1A,B). However, some discrepancies were observed between the relative abundances of *Acinetobacter baumannii, Mycoplasma* spp., *Pseudomonas aeruginosa*, and *Streptococcus* spp. in Pt [04] samples. Overall, a significant correlation was observed between V1-V2 and mixed V1-V2/ITS2 primers (Figure 1C).

**Figure 2:**
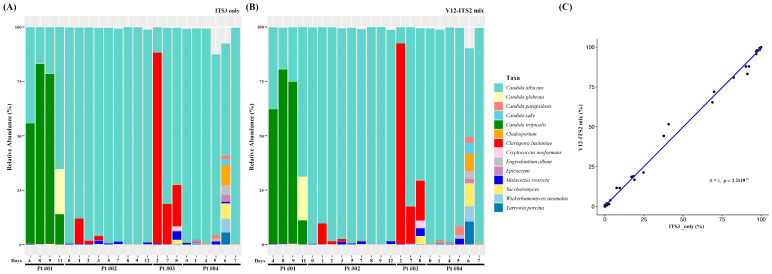
Relative abundances of fungal species depending on primers: (**A**) ITS2, (**B**) V1-V2/ITS2 primers, and (**C**) the correlation between abundances determined using ITS2 and V1-V2/ITS2 primers.

Next, the relative abundances of fungal communities, as determined using ITS2 primers and mixed V1-V2/ITS2 primers, were compared. When the relative abundances were plotted for individual samples for specific primers, most of the taxa showed similar abundances (Figure 2A,B). The correlation between abundances determined using ITS2 and mixed V1-V2/ITS2 primers was significant (Figure 2C). Taken together, the results obtained using the V1-V2/ITS2 primers, V1-V2, and ITS2 primers were comparable, indicating that the V1-V2/ITS2 primers simultaneously produced a relative abundance similar to those obtained using individual single primers.

### 3.3. Microbial DNA Standard

**Figure 3:**
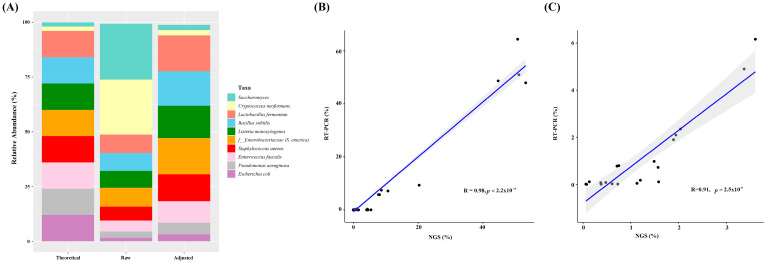
Adjustment of relative abundance. Read counts of taxa assigned to the fungi kingdom were reduced by a factor of 20 for adjustment. (**A**) Relative abundances in the microbial community DNA standard. Fungal relative abundances were adjusted to match abundances in the microbial community DNA standard. (**B**) Correlation between relative bacterial abundances determined by RT-PCR and V1-V2/ITS2 targeting NGS after adjusting fungal abundance. (**C**) Correlation between *Candida albicans* relative abundance determined by RT-PCR and V1-V2/ITS2 targeting NGS after adjusting fungal abundance. RT-PCR, real-time PCR; NGS, next-generation sequencing.

To test how well V1-V2/ITS2 primers performed in terms of predicting mixed bacterial and fungal abundances, we used a microbial community DNA standard containing a mixture of genomic DNA isolated from pure cultures of eight bacterial and two fungal strains. When relative abundances were plotted versus raw read counts, determined relative abundances of the two fungal strains almost reached 50%, which compared with a theoretical abundance of less than 5%. Thus, read counts of taxa assigned to the fungi kingdom were reduced by a factor of 20. For example, if the initial read count for the fungi kingdom was 20,000 and the read count for the bacteria kingdom was 100,000, the taxa assigned to the fungi kingdom was reduced to 1000. After adjustment, predicted relative abundances closely matched theoretical abundance (Figure 3A).

Using adjusted relative abundances, we examined the correlation between V1-V2/ITS2 targeting next-generation sequencing (NGS) and real-time PCR (RT-PCR). Bacterial (Figure 3B) and *C. albicans* (Figure 3C) abundances showed significant correlations indicating the adjustment made optimally predicted abundances in clinical samples.

### 3.4. Alpha Diversity Depending on Primers

**Figure 4:**
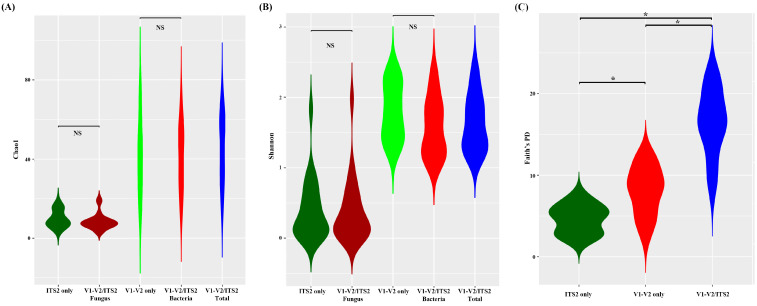
Microbiome alpha diversities determined using the three primer systems: (**A**) Chao1, (**B**) Shannon, and (**C**) Faith’s phylogenetic diversity (Faith’s PD) was determined for each primer. Differences were assessed using the Mann–Whitney U test. * indicates a *p*-value < 0.05. NS, not significant.

Chao1, Faith PD, and Shannon indices were used to evaluate alpha diversities depending on the primers. Similar Chao1 and Shannon index was observed between V1-V2 and bacterial reads from mixed V1-V2/ITS2 primers and between ITS2 and fungal reads from mixed V1-V2/ITS2 primers (Figure 4A,B). However, V1-V2/ITS2 primers exhibited significantly higher richness than ITS2 or V1-V2 primers based on Faith PD results, which incorporate phylogenetic differences (Figure 4C).

### 3.5. Simultaneous Analysis of Bacterial and Fungal Communities

**Figure 5:**
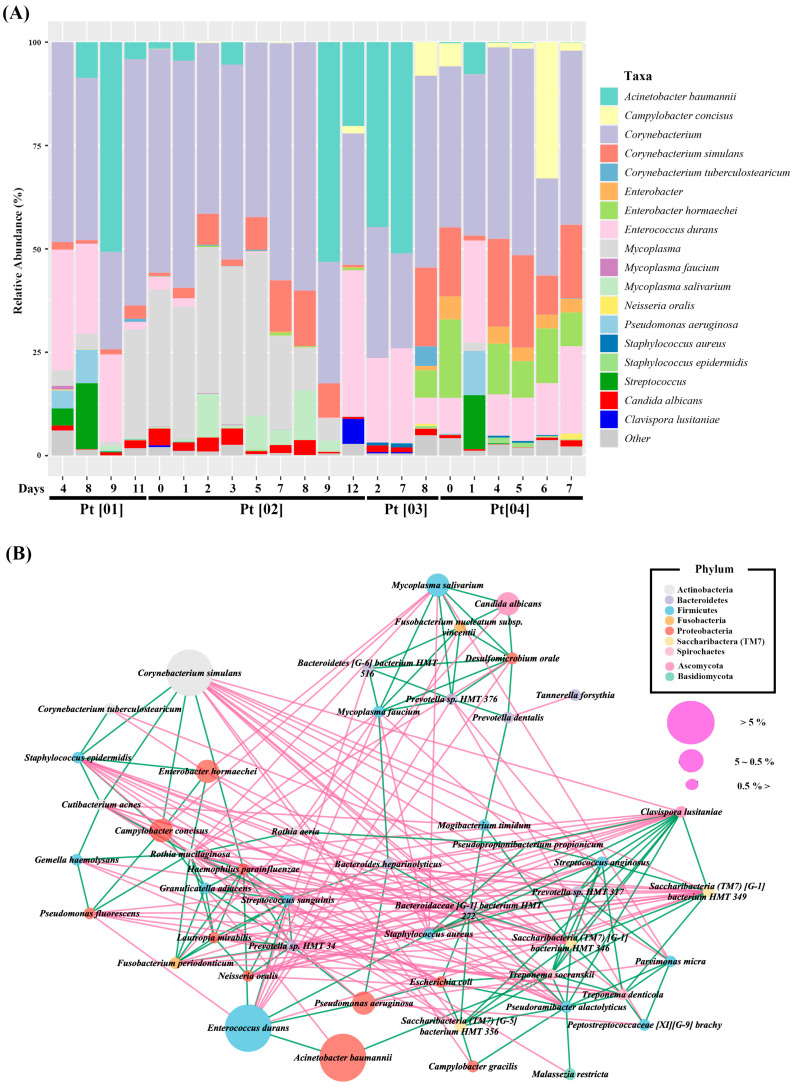
Simultaneous analysis of bacterial and fungal communities in buccal samples using the mixed V1-V2/ITS2 primer primers. (**A**) Relative abundances at the species level as determined using V1-V2/ITS2 primer. Relative abundances were plotted for each sample. (**B**) Microbiome network. Bubbles represent species, and colors indicate phyla as described in the legend. Connections between bubbles indicate abundances were correlated. Green and red lines represent positive and negative correlations, respectively. Bubble sizes represent relative abundances.

Next, bacterial and fungal relative abundances determined using the mixed V1-V2/ITS2 primers were simultaneously plotted for individual samples (Figure 5A). In intubated patients, the mean relative abundances of *C. albicans* and *Clavispora lusitaniae* were 1.46% ± 1.26% (max: 4.05%) and 0.33% ± 1.25% (max: 6.05%), respectively.

Finally, a microbiome network was constructed and showed highly dense inter- and intrakingdom interactions (Figure 5B). Interestingly, *C. albicans* showed positive correlations with several bacteria, including *Mycoplasma salivarium*, *Fusobacterium nucleatum* subsp. *Vincentii*, and *Desulfomicrobium orale*. In addition, *C. lustitaniae* formed a dense network with oral bacteria and its abundances were positively correlated with those of *Treponema socranskii*, *Staphylococcus aureus*, *Saccharibacteria (TM7)* spp., and *Streptococcus anginosus* and negatively correlated with *Rothia mucilaginosa*, *Haemophilus parainfluenzae*, *Lautropia mirabilis*, *Streptococcus sanguinis*, and *Neisseria oralis*.

## 4. Discussion

The human oral microbiome is a most diverse ecosystem, comprising numerous communities [26]. In this ecosystem, more than 600 bacterial [27] and 100 fungal species [28] compose the microbiome. Microbes residing in the human oral cavity have been well-reflected in human health and disease [29]. However, the oral fungal microbiome, also known as mycobiome, has not received as much scrutiny despite its ecological and clinical importance [30], as evidenced by its relative lack of attention [28]. As a result, potential interactions between the two kingdoms in the oral microbiome have yet to be thoroughly investigated. To gain a complete understanding of how the human oral microbiota affects health and disease, it is essential to take a comprehensive approach that accounts for both intra- and cross-kingdom interactions within the oral microbiota [31].

The use of high-throughput sequencing for 16S/ITS targeted amplicon high-throughput sequencing and metagenomics has allowed for a closer examination of the bacterial aspect of the human microbiome to better comprehend its function in health and disease [32,33]. The simultaneous detection of bacteria and fungi communities has several advantages over sequencing individual kingdoms. Simultaneous detection can provide relative abundance in the two kingdoms and enable the identification of intra- and interkingdom interactions. In this study, we sought to determine whether the abundances of bacterial and fungal communities can be simultaneously determined using primers targeting the bacterial V1-V2 region and the fungal ITS2 region.

Total read counts obtained using mixed V1-V2/ITS2 primers were significantly higher than those obtained using V1-V2 or ITS2 primers (Table). Since all samples were run simultaneously and equal concentrations of libraries were applied to chips, it is not clear why the V1-V2/ITS2 primer produced significantly higher read counts. Furthermore, the input to nonchimeric read count percentage was significantly higher for the ITS2 primer than the V1-V2 or V1-V2/ITS2 primers. Since all the samples were worked up using the same PCR conditions, PCR cycles are probably not responsible for these differences. On the other hand, primer specificities or target DNA abundances within samples might explain these differences [34].

After preprocessing, the relative abundances of bacterial and fungal communities were compared to determine whether mixing primers influenced results (Figure 1 and Figure 2). When relative abundances obtained using the different primers were plotted for individual samples, most taxa showed similar abundances. However, some discrepancies in the relative abundances of bacteria were observed at the species level in some samples. Nonetheless, when the relative abundances of assigned taxa were compared, high correlations were observed for bacterial and fungal communities. Taken together, mixed V1-V2/ITS2, V1-V2, and ITS2 primers produced comparable results, suggesting that the use of V1-V2/ITS2 primers simultaneously produced relative abundances similar to those obtained using individual single primers.

Next, a microbial DNA standard was used to test the ability of the V1-V2/ITS2 primer mix to simultaneously predict the abundance of bacterial and fungal species (Figure 3). When we plotted relative abundances versus raw read counts, large differences were found between theoretical and predicted abundances. We suggest several possible reasons for this discrepancy. First, the copy numbers of 16S and 18S rRNA for bacteria and fungi are markedly different. For bacteria, the average 16S rRNA copy number is 4.2 [35], while the 18S rRNA copy number for fungi ranges from 38 to 91 [36], and for *C. albicans*, the copy number of 18S rRNA is 55 [37], which suggests the use of 18S rRNA exaggerated the abundance of fungi. Second, ITS2 primers produced more nonchimeric reads, which would also increase the relative abundance of fungi. Thus, adjustment was required for the fungal strains. When NGS and RT-PCR abundances were plotted using adjusted relative abundance, bacterial and *C. albicans* abundances showed significant correlations, which indicated the adjustment optimally predicted bacterial and fungal abundances in clinical samples.

Finally, we plotted bacterial and fungal relative abundances obtained using mixed V1-V2/ITS2 primers. When alpha diversity was determined, a similar Chao1 and Shannon index was observed between V1-V2 and bacterial reads from mixed V1-V2/ITS2 primers and between ITS2 and fungal reads from mixed V1-V2/ITS2 primers (Figure 4A,B). These results support that the mixed V1-V2/ITS2 primer produced results comparable to those produced by individual V1-V2 and ITS2 primers, respectively. Since fungal diversity was significantly lower than bacterial diversity, no difference was observed between V1-V2 and V1-V2/ITS2 primers, suggesting that fungal diversity was completely masked by bacterial diversity. However, V1-V2/ITS2 primers exhibited significantly greater richness than the ITS2 or V1-V2 primers based on Faith PD results, which incorporate phylogenetic differences (Figure 4C). Thus, Faith’s PD seems to save fungal diversity information.

In many studies, the correlations between the abundances of fungal and bacterial species have been analyzed following individual by community analysis [38,39,40]. Furthermore, since information about the relative abundances of bacteria and fungi is limited, it is difficult to merge reported relative abundances directly in a single plot. When we simultaneously plotted bacterial and fungal relative abundances obtained using V1-V2/ITS2 primers for individual samples, the relative abundances of *C. albicans* and *Clavispora lusitaniae* were determinable within bacterial populations (Figure 5A). One of the advantages of using V1-V2/ITS2 primers is that results enable shifts in fungal relative abundances to be better understood than those obtained using individual primers. For example, in a patient [01], an increase in *C. tropicalis* abundance was observed on days 8 and 9 samples (Figure 2). However, when V1-V2/ITS2 primers were used, the overall relative abundance of fungus was found to decrease on days 8 and 9, indicating the increases in *C. tropicalis* abundance observed on these days were the result of *C. tropicalis* surviving during a decrease in total fungal abundance rather an increase in *C. tropicalis* abundance (Figure 5). Thus, the simultaneous determination of bacterial and fungal community abundances provides more comprehensive information regarding microbiome changes compared to single analysis.

A microbiome network was constructed to visualize bacterial and fungal interactions and revealed strong inter- and intrakingdom interactions (Figure 5B). Interestingly, *C. albicans* interacted positively with *Mycoplasma salivarium*, *Fusobacterium nucleatum* subsp. *Vincentii*, and *Desulfomicrobium orale* bacteria. There are many studies on interactions between Candida and oral bacteria, such as *C. albicans* and *S. mutans* [41,42], which have influenced their synergistic relationship to oral health. It is a very useful study model that could serve as a preventive or therapeutic target for oral diseases. However, the abundance of *S. mutans* is relatively low in the oral samples of healthy elderly subjects [43], and *S. mutans* is rarely found in intubated patients [44]. Thus, other oral bacterial species should also be considered in studies on interactions between bacteria and *C. albicans*. In the present study, *C. lustitaniae* formed a dense network with several oral bacteria; for example, its abundance was positively correlated with those of *Treponema socranskii, Staphylococcus aureus, Saccharibacteria (TM7)* spp., and *Streptococcus anginosus* and negatively correlated with those of *Rothia mucilaginosa, Haemophilus parainfluenzae, Lautropia mirabilis, Streptococcus sanguinis,* and *Neisseria oralis.* In a previous study, *Rothia*, *Haemophilus*, *Lautropia*, and *Neisseria* were more frequently found in healthy individuals, whereas *Treponema, Staphylococcus,* and *Saccharibacteria (TM7)* were more frequent under disease conditions, such as periodontitis [45]. The biological relevance of these correlations has not been determined, and thus, studies are required to determine the nature of the relationships between fungal and bacterial species, as they may reveal interactions that enable modulation of the relative abundances of targeted species.

This study is limited by a relatively small sample size caused by the requirement for family consent for the collection of oral samples from ICU patients. Additionally, only elderly patients were included, which could have introduced bias due to the limited number of participants. Therefore, we recommend a larger, well-designed study be undertaken to validate our findings in the future.

In the bacterial and fungal microbiome analysis, data regarding technical issues related to primer development are still lacking. In this study, we demonstrated that analyzing bacterial and fungal communities simultaneously in human oral samples can reduce analysis costs and enable the identification of infectious causes across two kingdoms. By performing network analysis, we suggested that bacterial–fungal interactions are present in the oral microbiome. Moreover, we identified *C. lustitaniae* and *C. albicans*, among a few other taxa, as potential keystone taxa of the oral microbiome of ICU patients. Overall, our study has facilitated an understanding of the determining factors and cross-kingdom interactions of the human oral microbiome. These findings will lead to the development of more effective approaches for diagnosing complex clinical infections. 

## Figures and Tables

**Table 1:** Numbers of sequence read counts during data preprocessing.

	ITS2 Only	V12 Only	V12-ITS2 Mix
Input read count	49,053 ± 40,833 (5826–195,379)	65,299 ± 61,068 (11,634–244,018)	127,081 ± 58,264 (41,789–267,196)
Filtered read count	44,991 ± 36,425	54,181 ± 50,877	107,511 ± 50,205
Denoised read count	44,881 ± 36,398	53,864 ± 50,718	107,117 ± 50,084
Merged read count	44,669 ± 36,114	52,796 ± 49,631	105,891 ± 49,384
Nonchimeric read count	43,640 ± 33,073 (4848–149,048)	47,743 ± 47,743 (8696–164,939)	98,815 ± 45,122 (31,587–195,606)
Percentage of input nonchimeric (%)	90.3 ± 3.7	73.8 ± 4.3	77.8 ± 4.3

## Data Availability

Data for this study, though not available in a public repository, will be made available upon reasonable request.

## Supplementary Materials

The following supporting information can be downloaded at: https://www.mdpi.com/article/10.3390/diagnostics13101784/s1, Table S1. Characterization of intubated subjects under mechanical ventilation, Table S2. Primers for quantification of bacteria and *Candida albicans*.

## References

[1] Brennan M.T., Bahrani-Mougeot F., Fox P.C., Kennedy T.P., Hopkins S., Boucher R.C., Lockhart P.B. (2004). The role of oral microbial colonization in ventilator-associated pneumonia. Oral Surg. Oral Med. Oral Pathol. Oral Radiol. Endod..

[2] Rello J., Diaz E. (2003). Pneumonia in the intensive care unit. Crit. Care Med..

[3] Kim S.H., Nah H.S., Kim J.B., Kim C.H., Kim M.S. (2021). Relationships Between Oral-Mucosal Pressure Ulcers, Mechanical Conditions, and Individual Susceptibility in Intubated Patients Under Intensive Care: A PCR-Based Observational Study. Biol. Res. Nurs..

[4] Chi H.W., Yang Y.S., Shang S.T., Chen K.H., Yeh K.M., Chang F.Y., Lin J.C. (2011). Candida albicans versus non-albicans bloodstream infections: The comparison of risk factors and outcome. J. Microbiol. Immunol. Infect..

[5] Krause R., Halwachs B., Thallinger G.G., Klymiuk I., Gorkiewicz G., Hoenigl M., Prattes J., Valentin T., Heidrich K., Buzina W. (2016). Characterisation of candida within the mycobiome/microbiome of the lower respiratory tract of ICU patients. PLoS ONE.

[6] Mencarini J., Mantengoli E., Tofani L., Riccobono E., Fornaini R., Bartalesi F., Corti G., Farese A., Pecile P., Boni L. (2018). Evaluation of candidemia and antifungal consumption in a large tertiary care Italian hospital over a 12-year period. Infection.

[7] Strollo S., Lionakis M.S., Adjemian J., Steiner C.A., Prevots D.R. (2016). Epidemiology of hospitalizations associated with invasive candidiasis, united states, 2002–2012. Emerg. Infect. Dis..

[8] Mehta Y., Gupta A., Todi S., Myatra S.N., Samaddar D.P., Patil Y., Bhattacharya P.K. (2014). Guidelines for prevention of hospital acquired infections. Indian J. Crit. Care Med..

[9] Sharma V.K., Mehta V., Singh T.G. (2020). Alzheimer’s Disorder: Epigenetic Connection and Associated Risk Factors. Curr. Neuropharmacol..

[10] Yin Y., Hountras P., Wunderink R.G. (2017). The microbiome in mechanically ventilated patients. Curr. Opin. Infect. Dis..

[11] Patangia D.V., Ryan C.A., Dempsey E., Ross R.P., Stanton C. (2022). Impact of antibiotics on the human microbiome and consequences for host health. Microbiol. Open.

[12] Tuon F.F., Gavrilko O., Almeida S., Sumi E.R., Alberto T., Rocha J.L., Rosa E.A. (2017). Prospective, randomised, controlled study evaluating early modification of oral microbiota following admission to the intensive care unit and oral hygiene with chlorhexidine. J. Glob. Antimicrob. Resist..

[13] Peterson J., Garges S., Giovanni M., McInnes P., Wang L., Schloss J.A., Bonazzi V., McEwen J.E., Wetterstrand K.A., Deal C. (2009). The NIH human microbiome project. Genome Res..

[14] Chakravorty S., Helb D., Burday M., Connell N., Alland D.A. (2007). detailed analysis of 16s ribosomal RNA gene segments for the diagnosis of pathogenic bacteria. J. Microbiol. Methods.

[15] Turenne C.Y., Sanche S.E., Hoban D.J., Karlowsky J.A., Kabani A.M. (1999). Rapid identification of fungi by using the its2 genetic region and an automated fluorescent capillary electrophoresis system. J. Clin. Microbiol..

[16] The International Council for Harmonization of Technical Requirements for Pharmaceuticals for Human Use. https://www.ema.europa.eu/en/glossary/guideline.

[17] Bonham P.A. (2009). Swab cultures for diagnosing wound infections: A literature review and clinical guideline. J. Wound Ostomy Cont. Nurs..

[18] Kozich J.J., Westcott S.L., Baxter N.T., Highlander S.K., Schloss P.D. (2013). Development of a dual-index sequencing strategy and curation pipeline for analyzing amplicon sequence data on the miseq illumine sequencing platform. Appl. Environ. Microbiol..

[19] Ihrmark K., Bodeker I.T., Cruz-Martinez K., Friberg H., Kubartova A., Schenck J., Strid Y., Stenlid J., Brandstrom-Durling M., Clemmensen K.E. (2012). New primers to amplify the fungal ITS2 region--evaluation by 454-sequencing of artificial and natural communities. FEMS Microbiol. Ecol..

[20] Fujita S.I., Senda Y., Nakaguchi S., Hashimoto T. (2001). Multiplex pcr using internal transcribed spacer 1 and 2 regions for rapid detection and identification of yeast strains. J. Clin. Microbiol..

[21] Nilsson R.H., Larsson K.H., Taylor A.F.S., Bengtsson-Palme J., Jeppesen T.S., Schigel D., Kennedy P., Picard K., Glockner F.O., Tedersoo L. (2019). The UNITE database for molecular identification of fungi: Handling dark taxa and parallel taxonomic classifications. Nucleic Acids Res..

[22] Hall M., Beiko R.G. (2018). 16s rrna gene analysis with qiime2. Methods Mol. Biol..

[23] Wade W.G. (2013). The oral microbiome in health and disease. Pharmacol. Res..

[24] Friedman J., Alm E.J. (2012). Inferring correlation networks from genomic survey data. PLoS Comput. Biol..

[25] Shannon P., Markiel A., Ozier O., Baliga N.S., Wang J.T., Ramage D., Amin N., Schwikowski B., Ideker T. (2003). Cytoscape: A software environment for integrated models of biomolecular interaction networks. Genome Res..

[26] Bassis C.M., Erb-Downward J.R., Dickson R.P., Freeman C.M., Schmidt T.M., Young V.B., Beck J.M., Curtis J.L., Huffnagle G.B. (2015). Analysis of the Upper Respiratory Tract Microbiotas as the Source of the Lung and Gastric Microbiotas in Healthy Individuals. mBio.

[27] Dewhirst F.E., Chen T., Izard J., Paster B.J., Tanner A.C., Yu W.H., Lakshmanan A., Wade W.G. (2010). The human oral microbiome. J. Bacteriol..

[28] Ghannoum M.A., Jurevic R.J., Mukherjee P.K., Cui F., Sikaroodi M., Naqvi A., Gillevet P.M. (2010). Characterization of the oral fungal microbiome (mycobiome) in healthy individuals. PLoS Pathog..

[29] Correa J.D., Fernandes G.R., Calderaro D.C., Mendonca S.M.S., Silva J.M., Albiero M.L., Cunha F.Q., Xiao E., Ferreira G.A., Teixeira A.L. (2019). Oral microbial dysbiosis linked to worsened periodontal condition in rheumatoid arthritis patients. Sci. Rep..

[30] Diaz P.I., Dongari-Bagtzoglou A. (2021). Critically appraising the significance of the oral mycobiome. J. Dent. Res..

[31] Baker J.L., Bor B., Agnello M., Shi W., He X. (2017). Ecology of the oral microbiome: Beyond bacteria. Trends Microbiol..

[32] Botterel F., Angebault C., Cabaret O., Stressmann F.A., Costa J.-M., Wallet F., Wallaert B., Bruce K., Dalhaes L. (2017). Fungal and Bacterial Diversity of Airway Microbiota in Adults with Cystic Fibrosis: Concordance Between Conventional Methods and Ultra-Deep Sequencing, and Their Practical use in the Clinical Laboratory. Mycopathologia.

[33] Delhaes L., Monchy S., Frealle E., Hubans C., Salleron J., Leroy S., Prevotat A., Wallet F., Wallaert B., Dei-Cas E. (2012). The Airway Microbiota in Cystic Fibrosis: A Complex Fungal and Bacterial Community—Implications for Therapeutic Management. PLoS ONE.

[34] Omelina E.S., Ivankin A.V., Letiagina A.E., Pindyurin A.V. (2019). Optimized PCR conditions minimizing the formation of chimeric DNA molecules from MPRA plasmid libraries. BMC Genom..

[35] Vetrovsky T., Baldrian P. (2013). The variability of the 16s rrna gene in bacterial genomes and its consequences for bacterial community analyses. PLoS ONE.

[36] Herrera M.L., Vallor A.C., Gelfond J.A., Patterson T.F., Wickes B.L. (2009). Strain-dependent variation in 18s ribosomal DNA copy numbers in aspergillus fumigatus. J. Clin. Microbiol..

[37] Skrzypek M.S., Binkley J., Binkley G., Miyasato S.R., Simison M., Sherlock G. (2017). The candida genome database (CGD): Incorporation of assembly 22, systematic identifiers and visualization of high throughput sequencing data. Nucleic Acids Res..

[38] Nash A.K., Auchtung T.A., Wong M.C., Smith D.P., Gesell J.R., Ross M.C., Stewart C.J., Metcalf G.A., Muzny D.M., Gibbs R.A. (2017). The gut mycobiome of the human microbiome project healthy cohort. Microbiome.

[39] Arfken A.M., Frey J.F., Summers K.L. (2020). Temporal dynamics of the gut bacteriome and mycobiome in the weanling pig. Microorganisms.

[40] Keum H.L., Kim H., Kim H.J., Park T., Kim S., An S., Sul W.J. (2020). Structures of the skin microbiome and mycobiome depending on skin sensitivity. Microorganisms.

[41] Koo H., Bowen W.H. (2014). Candida albicans and streptococcus mutans: A potential synergistic alliance to cause virulent tooth decay in children. Future Microbiol..

[42] Ellepola K., Truong T., Liu Y., Lin Q., Lim T.K., Lee Y.M., Cao T., Koo H., Seneviratne C.J. (2019). Multi-omics analyses reveal synergistic carbohydrate metabolism in streptococcus mutans-candida albicans mixed-species biofilms. Infect. Immun..

[43] Hallang S., Esberg A., Haworth S., Johansson I. (2021). Healthy oral lifestyle behaviours are associated with favourable composition and function of the oral microbiota. Microorganisms.

[44] Sands K.M., Twigg J.A., Lewis M.A.O., Wise M.P., Marchesi J.R., Smith A., Wilson M.J., Williams D.W. (2016). Microbial profiling of dental plaque from mechanically ventilated patients. J. Med. Microbiol..

[45] Ohara-Nemoto Y., Haraga H., Kimura S., Nemoto T.K. (2008). Occurrence of staphylococci in the oral cavities of healthy adults and nasal oral trafficking of the bacteria. J. Med. Microbiol..

