# Supplementary material for: Simultaneous Analysis of Bacterial and Fungal Communities in Oral Samples from Intubated Patients in Intensive Care Unit
Source: Diagnostics (Basel). 2023 May 18;13(10):1784. doi: 10.3390/diagnostics13101784 (PMC10217707; doi:10.3390/diagnostics13101784)
Supplement: Supplementary file 1 [file diagnostics-13-01784-s001.zip › diagnostics-2331692-supplementary.pdf]

Supplementary Table

Table S1. Characterization of intubated subjects under mechanical ventilation

Subject	Age	Sex	Underlying disease	Initial Antibiotics
#pt-01	81	M	COPD*, Alcoholic hepatitis, Diabetes	Piperacillin-Tazobactam
#pt-02	71	M	Lung Cancer, Renal Cell Cancer	Meropenem
#pt-03	67	M	Alcoholic Hepatitis	Ertapenem
#pt-04	65	M	Diabetes, COPD*	Piperacillin-Tazobactam

* COPD: Chronic obstructive pulmonary disease

Table S2. Primers for quantification of bacteria and *Candida albicans*.

Primer	Sequence (5' – 3')
Acinetobacter baumannii	
**Abau1**	GGCTTATTAAGTCGGATGTG
**Abau2**	ACCTCAGCGTCAGTATTAG
Pseudomonas aeruginosa	
**Psae1**	TTCAGCAAGTTGGATGTG
**Psae2**	CCTCAGTGTCAGTATCAGT
Staphylococcus aureus	
**Saur1**	TGCATTAGCTAGTTGGTAAG
**Saur2**	ATATGTTCTTCCCTAATAACAGAG
Staphylococcus epidermidis	
**Sepi1**	CCGCATTAGCTAGTTGGTA
**Sepi2**	CTTACACATTTGTTCTTCCCTA
Candida albicans	
**Calb1**	TTTATCAACTTGTCACACCAGA
**Calb2**	ATCCCGCCTTACCACTACCG
16S rRNA	
**16S_1**	CCTACGGGAGGCAGCAG
**16S_2**	ATTACCGCGGCTGCTGGCA
